# Salicylic Acid Accumulation Controlled by LSD1 Is Essential in Triggering Cell Death in Response to Abiotic Stress

**DOI:** 10.3390/cells10040962

**Published:** 2021-04-20

**Authors:** Maciej Jerzy Bernacki, Anna Rusaczonek, Weronika Czarnocka, Stanisław Karpiński

**Affiliations:** 1 Institute of Technology and Life Sciences, Falenty, Al. Hrabska 3, 05-090 Raszyn, Poland; mjbernacky@gmail.com; 2 Department of Botany, Institute of Biology, Warsaw University of Life Sciences, Nowoursynowska Street 159, 02-776 Warsaw, Poland; anna_rusaczonek@sggw.edu.pl (A.R.); weronika_czarnocka@sggw.edu.pl (W.C.); 3 Department of Plant Genetics, Breeding and Biotechnology, Institute of Biology, Warsaw University of Life Sciences, Nowoursynowska Street 159, 02-776 Warsaw, Poland

**Keywords:** abiotic stress, cell death, LESION SIMULATING DISEASE 1, salicylic acid, UV

## Abstract

Salicylic acid (SA) is well known hormonal molecule involved in cell death regulation. In response to a broad range of environmental factors (e.g., high light, UV, pathogens attack), plants accumulate SA, which participates in cell death induction and spread in some foliar cells. LESION SIMULATING DISEASE 1 (LSD1) is one of the best-known cell death regulators in *Arabidopsis thaliana*. The *lsd1* mutant, lacking functional LSD1 protein, accumulates SA and is conditionally susceptible to many biotic and abiotic stresses. In order to get more insight into the role of LSD1-dependent regulation of SA accumulation during cell death, we crossed the *lsd1* with the *sid2* mutant, caring mutation in *ISOCHORISMATE SYNTHASE 1* *(ICS1*) gene and having deregulated SA synthesis, and with plants expressing the bacterial *nahG* gene and thus decomposing SA to catechol. In response to UV A+B irradiation, the *lsd1* mutant exhibited clear cell death phenotype, which was reversed in *lsd1/sid2* and *lsd1*/*NahG* plants. The expression of *PR*-genes and the H_2_O_2_ content in UV-treated *lsd1* were significantly higher when compared with the wild type. In contrast, *lsd1/sid2* and *lsd1*/*NahG* plants demonstrated comparability with the wild-type level of *PR*-genes expression and H_2_O_2_. Our results demonstrate that SA accumulation is crucial for triggering cell death in *lsd1*, while the reduction of excessive SA accumulation may lead to a greater tolerance toward abiotic stress.

## 1. Introduction

In their natural environment plants are constantly and simultaneously exposed to many biotic and abiotic environmental factors, such as various pathogens, excess/deficiency of light, UV irradiation, drought, chilling, heat, and salinity. More and more data clearly indicate that plants have developed molecular and genetic systems to simultaneously respond to a mixture of biotic and abiotic stress factors [1,2,3,4,5]. One of the mechanisms important in plants’ response to stress is programmed cell death (PCD). PCD is a very sophisticated and selective molecular and physiological process leading to the death of some cells [1,6,7,8,9], which triggers a beneficial immune defense and acclimatory response in others [10,11,12,13,14].

Many PCD regulatory proteins in *Arabidopsis thaliana* have been described, but one of the best known is LESION SIMULATING DISEASE 1 (LSD1). LSD1 encodes a small C2C2 zinc finger protein that is a negative regulator of PCD [15], playing a molecular function of transcriptional regulator and scaffold protein [16]. It was shown that *lsd1* mutant plants depleted in LSD1 functional protein are very susceptible to biotic stresses [17] and to abiotic stresses such as high light [18], chilling [19], and UV irradiation [1,5]. Importantly, LSD1 acts as a negative switch for two positive cell death regulators, ENHANCED DISEASE SUSCEPTIBILITY 1 (EDS1) and PHYTOALEXIN DEFICIENT 4 (PAD4) [16,20]. LSD1, EDS1, and PAD4 proteins form a specific hub that is responsible for triggering salicylic acid (SA)-, ethylene (ET)-, and reactive oxygen species (ROS)-dependent cell death and acclimatory responses to unfavorable conditions [1,20,21,22].

SA is one of the most important phytohormones in plant defense signaling [23]. It was shown that both biotic [17,24] and abiotic stresses [1,5] induce SA biosynthesis. Plants possess two different pathways to synthesize SA, both starting from chorismate. One of them is a pathway engaging isochorismate synthase (ICS), and the other is a pathway involving phenylalanine ammonia-lyase (PAL). The contribution of these two pathways is species-dependent. In *Oryza sativa* the PAL pathway is the most important in SA accumulation, while in Arabidopsis the ICS pathway prevails [25,26]. The ICS pathway starts from the conversion of chorismate into isochorismate (IC) by the ICS enzyme [27,28,29] and was first found in bacteria [30]. However, it was found that SA synthesis via the ICS pathway in *Arabidopsis thaliana* differs significantly from that in bacteria. The ICS pathway relies on amino acid conjugation of L-glutamate to IC, which is then spontaneously decomposed or enzymatically conversed, resulting in the formation of SA. The gene encoding enzyme responsible for this reaction, AVRPPHB SUSCEPTIBLE 3 (PBS3, AT5G13320), has been characterized in Arabidopsis but not in any other plant species so far [31,32]. The ICS pathway is very important in pathogen-induced SA accumulation [33] and in response to abiotic stress such as UV irradiation [34]. In Arabidopsis, two ICS homologs were found: *ICS1* and *ICS2*. The mutation in *ICS1* significantly lowers SA levels in response to UV stress, while the mutation in *ICS2* does not [35]. This suggests that *ICS1* is the main contributor to basal- and UV-induced SA accumulation in Arabidopsis [26], and the mutant in the *ICS1* gene is called *salicylic acid induction deficient 2* (*sid2*) [27].

It was shown that mutants with lower SA levels demonstrate better fitness, produce more seeds, and accumulate increased biomass [5,36], while mutants with higher SA content exhibit a dwarf phenotype [37,38]. Interestingly, some mutants with decreased SA biosynthesis are more susceptible to biotrophic pathogen infection (i.e., *enhanced disease susceptibility 5* (*eds5*) and *sid2*) [39,40], while others are more resistant to abiotic stresses (i.e., *eds1* or *pad4*) [5]. Findings indicate that the role of SA in response to biotic and abiotic stresses is different, and it was postulated that SA acts as double-edged sword for PCD in plants [41].

In response to stress, the *lsd1* mutant was proved to accumulate SA and to strongly exhibit the cell death phenotype in laboratory conditions but not in the field [5,42,43]. SA accumulation in *lsd1* is reverted in double *eds1/lsd1* and *pad4/lsd1* mutants, accumulating significantly less SA and ROS than the single *lsd1* mutant [5]. However, the role of SA in LSD1-, EDS1-, and PAD4-dependent PCD regulation has not been broadly studied in response to abiotic stresses. Therefore, in this study we aimed to check the relationship between the SA content and the LSD1/EDS1/PAD4-dependent PCD in response to UV A+B stress. We crossed the *lsd1* mutant with the *sid2* mutant or with a transgenic line expressing *nahG* (*NahG*) [27,36,44], which allowed us to conclude a role of SA in the regulation of PCD that is dependent on LSD1/EDS1/PAD4 during abiotic stress response.

## 2. Materials and Methods

### 2.1. Plant Material and Growth Conditions

**Figure 1:**
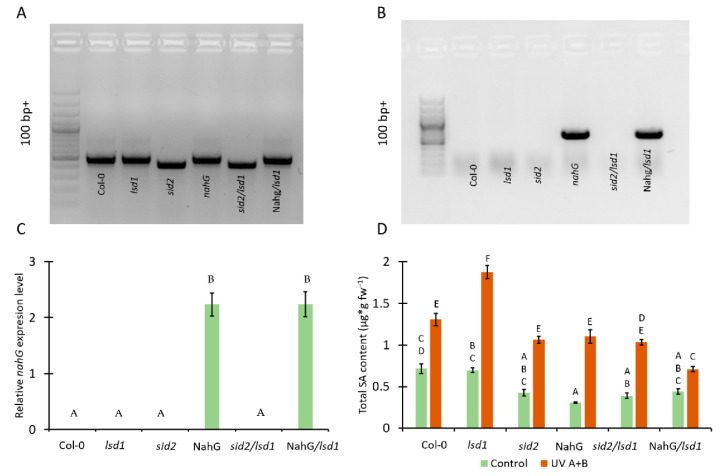
Genotyping of plant material used in this study and the effect of genetic background on the level of total salicylic acid (SA). (**A**) Detection of mutation in the *ICS1* gene using PCR, (**B**) detection of the presence of the *nahG* gene using PCR, (**C**) relative *nahG* expression level, (**D**) total SA content in tested plants before UV treatment (green bars) and after UV irradiation (2000 mJ·cm^−^^2^*)* (orange bars). Within a subgraph, values sharing common labels (letters) are not significantly different from each other (*p* > 0.001) (*n* = 9).

*Arabidopsis thaliana* plants (Col-0, *lsd1*, *sid2*, *NahG*, *sid2/lsd1* and *NahG*/*lsd1*) were grown in a walk in-type growing chamber (Siemens, München, Germany) under the following conditions: 8/16 h photoperiod, photosynthetic photon flux density of 80 μmol photons m^−2^·s^−1^, air humidity of 50%, and day/night temperature of 20/18 °C. The *sid2/lsd1* and *NahG*/*lsd1* plants were obtained by crossing. F3 generation was checked using PCR and qPCR (Figure 1A–C). Genotyping of *sid2* mutant was described previously [38]. Genotyping of *NahG* transgenic plants was performed using the following PCR condition: initialization 3 min 95 °C, 30-times repeated denaturation 30 s 95 °C, annealing 30 s 56 °C, elongation 40 s 72 °C, and final elongation 1 min 72 °C. Each PCR mixture included 13.9 µL water, 2 µL 10× buffer (Sigma-Aldrich, St. Louis, MO, USA), 2 µL deoxyribonucleotide triphosphate (2.5 mmol), 1 µL mix of forward and reverse primers (10 mmol), 0.1 µL DreamTaq DNA Polymerase (5 U/µL) (Sigma-Aldrich, St. Louis, MO, USA), and 1 µL of total genomic DNA. All primers used in this study are listed in Table 1. A band of approximately 500 bp was obtained for the *nahG* gene [38]. All experiments were performed on 4-week-old plants.

### 2.2. Ultraviolet Irradiation Application

For UV A+B stress application, the UV 500 Crosslinker (Hoefer Pharmacia Biotech, San Francisco, CA, USA) was used. It was equipped with three UV-B lamps (type G8T5E, Sankyo Denki, peak wavelength 306 nm) and two UV-A lamps (type TL8WBLB, Philips, peak wavelength 365 nm). Arabidopsis mutants were exposed to a single irradiation dose 2000 mJ·cm^−^^2^. All analyses described in this study were performed 24 h after stress application.

### 2.3. Relative Electrolyte Leakage Measurement

The Arabidopsis rosettes were cut and placed in 50 mL falcon tubes filled with 35 mL of Milli-Q water (Merc Millipore, Darmstadt, Germany). The relative electrolyte leakage was measured with a conductance meter pHenomenal^®^ CO 3100 L (VWR, Gdańsk, Poland) and calculated as a ratio between the value obtained after 1 h incubation and the total leakage evaluated after freezing the samples in −80 °C overnight followed by defrosting.

### 2.4. Trypan Blue Staining

Trypan blue (TB) stock (30 µmol trypan blue; Sigma-Aldrich, St. Louis, MO, USA) in a mixture of lactic acid, glycerol, and water (10 mL:10 mL:20 mL) was diluted with 96% ethanol (1:2) to obtain TB working solution. Fully developed leaves from non-treated plants and plants treated with UV A+B were collected and dipped immediately in TB working solution in 50 mL falcon tubes. Leaves were incubated in TB working solution for 30 min at room temperature and gently shaken several times. Subsequently, the TB working solution was removed and replaced which methanol. The leaves were incubated in methanol for 24 h, and methanol was changed several times for fresh methanol. Leaves deprived of chlorophyll were visualized using a Nikon SMZ18 stereomicroscope (Nikon Inc., Melville, NY, USA) with an adapted camera Nikon d5100 (Nikon Inc., Melville, NY, USA). Pictures of individual leaves were analyzed using ImageJ software version 1.8.0 (http://rsb.info.nih.gov/ij, accessed on 20 February 2021), and blue dots (micro-lesions) were counted per mm^2^ of leaf area.

### 2.5. RNA Isolation, cDNA Synthesis and qPCR Analysis

Arabidopsis rosettes were collected and immediately frozen in liquid nitrogen in three independent biological replicates, each containing 15–20 individual plants. Total RNA extraction was performed using a GeneMATRIX Universal RNA Purification Kit (EURX, Gdańsk, Poland) with an additional step of on-column DNaseI digestion. RNA concentration and purity were checked using an Eppendorf BioSpectrometer (Eppendorf, Hamburg, Germany). The RNA quality was controlled by electrophoretic separation in 1% agarose gel. cDNA synthesis was performed for equimolar RNA amounts of each sample using a High Capacity cDNA Reverse Transcription Kit (Thermo Fisher Scientific). qPCRs were performed in three technical repetitions for each of the three biological replicates using the Power SYBR Green PCR Master Mix and the ABI 7500 Fast Real-Time PCR System (Thermo Fisher Scientific, Waltham, Massachusetts, USA). Two reference genes were used: *5-FORMYLTETRAHYDROFOLATE CYCLOLIGASE* (*5-FCL*, AT5G13050) and *PROTEIN PHOSPHATASE 2A SUBUNIT A2* (*PP2AA2*, AT3G25800). Primers are listed in Table 1.

### 2.6. Measurement of SA and Hydrogen Peroxide (H_2_O_2_) Content and Ascorbate Peroxidase (APX) Activity

The methodology for measuring the content of SA, *H_2_O_2_*, and APX activity has been precisely described previously [42,45,46,47].

## 3. Results

### 3.1. Mutation in ICS1 and Expression of Bacterial Nahg Results in Lower Accumulation of SA in Lsd1 Mutant Background

In order to study the relationship between the SA content and LSD1/EDS1/PAD4-dependent response to UV A+B stress, the double mutant in *ICS1* and *LSD1* genes (*sid2/lsd1*) were obtained (Figure 1A). Moreover, the *lsd1* mutant expressing bacterial *nahG* gene (*NahG*/*lsd1*) was generated by crossing (Figure 1A,B). ICS1 protein is known as a crucial component in SA synthesis [24], while bacterial nahG protein decomposes SA to catechol [36].

Before UV treatment, we found no significant differences in SA content between Col-0, *lsd1*, *sid2*, and *NahG*/*lsd1*, while *sid2*/*lsd1* and *NahG* plants exhibited significantly lower levels of SA in their tissues. After UV irradiation, all tested genotypes accumulated more SA than before stress. The *sid2*, *NahG*, and *sid2*/*lsd1* did not differ in terms of SA level when compared with the Col-0, while *NahG*/*lsd1* accumulated significantly less SA than Col-0. However, the *lsd1* mutant showed the highest content of SA, much higher than Col-0 or other genotype. Importantly, after UV stress the *sid2*/*lsd1* and *NahG*/*lsd1* plants exhibited significantly lower SA content in comparison with the *lsd1* mutant (Figure 1D), which indicates that the mutations in *ICS1* or *nahG* expression are able to revert the *lsd1*-specific SA accumulation.

### 3.2. Lower Foliar SA Level Mitigates the Lsd1-Specific Cell Death Phenotype

**Figure 2:**
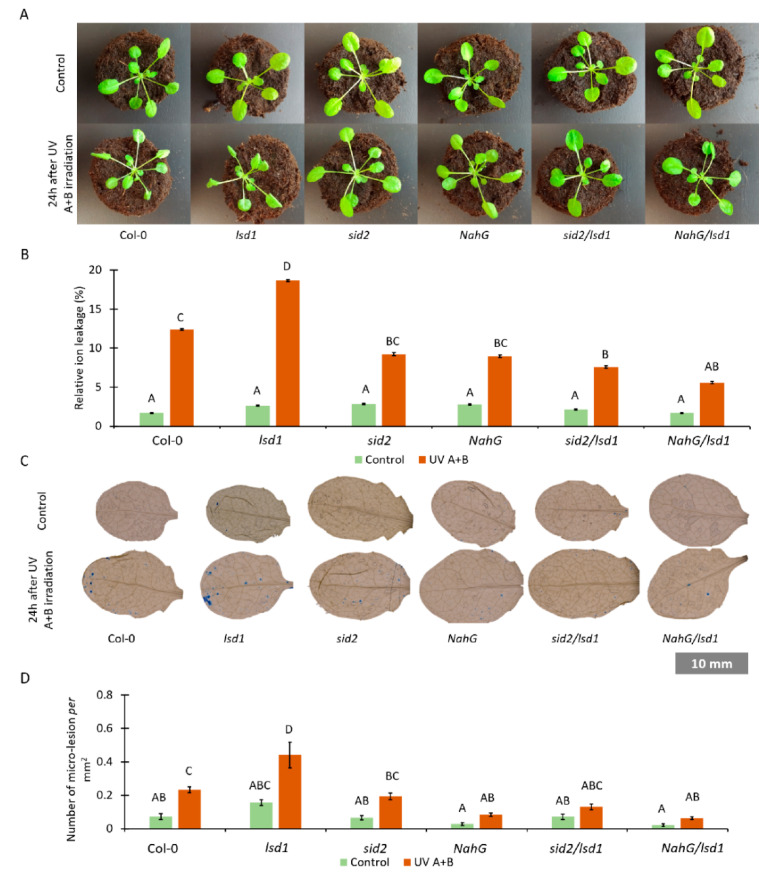
The effect of SA synthesis or metabolism deregulation on LSD1-regulated cell death. (**A**) Pictures of plants cultivated under laboratory non-stress conditions (top row) and 24 h after episode of UV irradiation (2000 mJ·cm^−^^2^) (bottom row), (**B**) relative ion leakage in plants before stress (green bars) and 24 h after UV irradiation (2000 mJ·cm^−^^2^) (orange bars), (**C**) trypan blue staining of dead cells in plants before stress (top row) and after UV irradiation (bottom row), and (**D**) cell death quantified as micro-lesion number per mm^2^ in plants before stress (green bars) and after UV irradiation (2000 mJ·cm^−^^2^) (orange bars). Within a subgraph, values sharing common labels (letters) are not significantly different from each other (*p* > 0.001) (*n* = 10–15).

SA is an important molecule in PCD regulation [48,49], and it is accumulated in the *lsd1* mutant [4,5]. In this work we wanted to know if deregulation in SA synthesis or metabolism can influence PCD in the *lsd1* background. We found no differences in the phenotype among tested genotypes grown under non-stress conditions (Figure 2A). However, 24 h after UV irradiation the PCD symptoms started to be visible (Figure 2A). The wild-type plants showed only subtle changes, such as twisted leaves. In the *lsd1* mutant, leaf curling was more visible. Other genotypes—*sid2*, *NahG*, *sid2*/*lsd1* and *NahG*/*lsd1*—did not exhibit apparent changes after UV irradiation.

In order to assess the level of cell death in tested genotypes, relative electrolyte leakage was measured. Before UV treatment we did not observe any differences among tested genotypes (Figure 2B). Twenty-four hours after UV irradiation, all tested genotypes exhibited higher ion leakage when compared with non-treated plants. Ion leakage was significantly higher in UV-treated *lsd1* when compared with the wild type (Figure 2B). However, *sid2*/*lsd1* and *NahG*/*lsd1* demonstrated significantly lower electrolyte leakage when compared with the *lsd1* background and with Col-0 plants.

**Figure 3:**
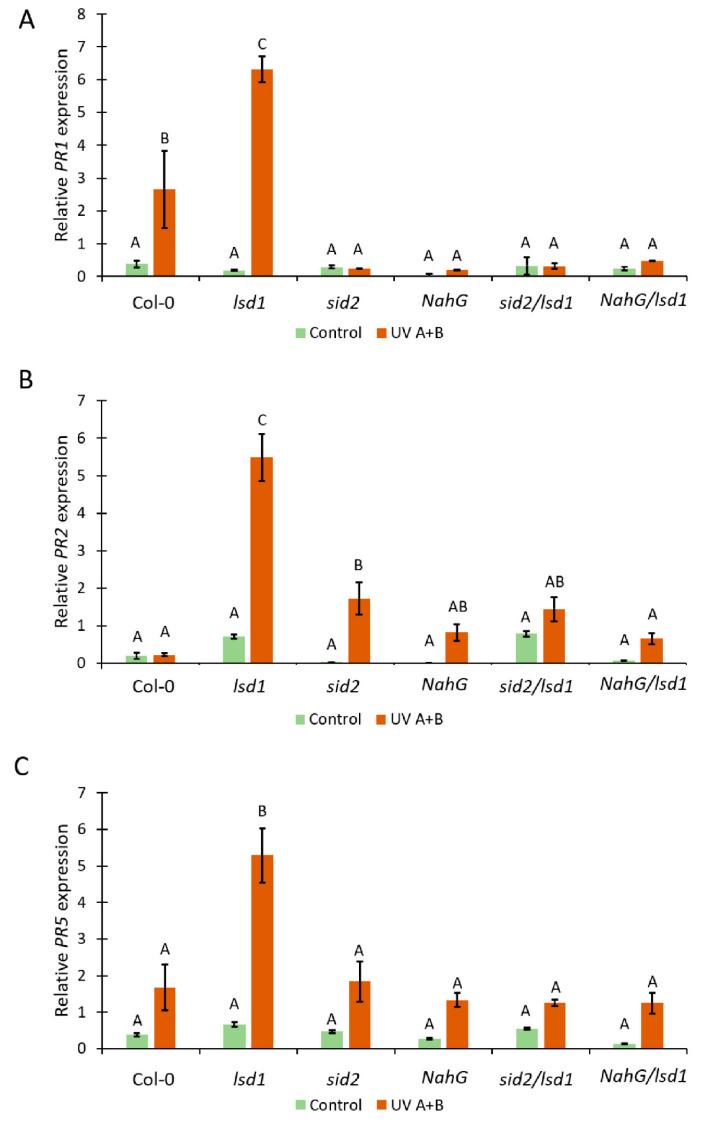
The effect of deregulation in SA synthesis or metabolism on *PR*-genes expression. Relative expression of (**A**) *PR1*, (**B**) *PR2*, and (**C**) *PR5* in tested genotypes before stress (green bars) and after UV irradiation (2000 mJ·cm^−^^2^) (orange bars). Within a subgraph, values sharing common labels (letters) are not significantly different from each other (*p* > 0.001) (*n* = 10–15). Results were normalized on two independent reference genes.

Moreover, micro-lesion formation using TB staining was checked. Micro-lesions constitute small lesion areas within the leaf tissue, comprising one or a couple of dead cells [50,51]. Before stress, we found no differences in the micro-lesion number among tested genotypes, while after UV irradiation in both the wild-type and *lsd1* mutant we found more micro-lesions than in plants with deregulated SA synthesis or metabolism (Figure 3C,D). All these results demonstrate that lower foliar concentration of SA mitigates the *lsd1*-specific cell death phenotype after stress.

### 3.3. Deregulated SA Synthesis or SA Decomposition Leads to Alterations in PR-Genes Expression

SA is involved in the induction of *PR*-genes expression [52]. Therefore, we decided to check the relative expression level of *PR1*, *PR2*, and *PR5* genes in genotypes tested within this study. Before stress, we found marginal but not statistically significant differences in the expression level of all tested *PR*-genes (Figure 3A–C). However, 24 h after the episode of UV irradiation in the wild type, the *PR1* expression was higher when compared with the plants before stress. This effect was significantly stronger in the *lsd1* mutant. The other tested Arabidopsis genotypes did not exhibit differences in *PR1* expression between control and stress conditions (Figure 3A). Importantly, *sid2*/*lsd1* and *NahG*/*lsd1* plants demonstrated significantly lower expression of *PR1* when compared with the single *lsd1* mutant or even with Col-0. The expression of *PR2* before stress did not differ between any tested genotypes. However, after UV irradiation, the expression of *PR2* in *lsd1* was almost 6 times higher than before stress. In the wild-type *NahG*, *sid2*/*lsd1*, and *NahG*/*lsd1*, there were no statistically significant increases in *PR2* expression after UV irradiation. Interestingly, we found slightly but significantly higher expression of *PR2* in *sid2* in comparison with wild-type plants (Figure 3B). The expression of *PR5* before stress and after UV irradiation followed a similar pattern as *PR2* expression. After UV treatment, only *lsd1* exhibited higher expression of *PR5*, while *sid2*/*lsd1* and *NahG*/*lsd1* had similar *PR5* expression as Col-0 (Figure 2C). This part of our research shows that lower SA content reverts the *lsd1*-specific high expression of PR-genes after stress.

### 3.4. Deregulation in the SA Synthesis or Metabolism Results in Changes in the Antioxidant System

**Figure 4:**
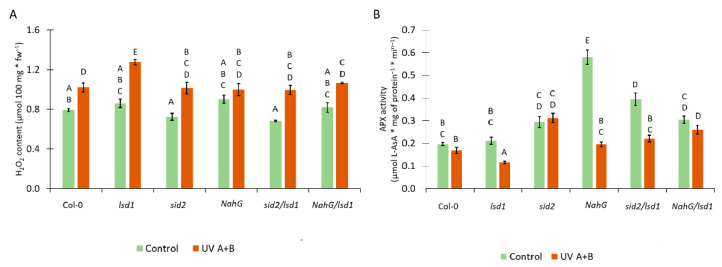
The influence of SA synthesis or metabolism deregulation on foliar redox status. (**A**) Foliar H_2_O_2_ content before stress (green bars) and 24 h after UV irradiation (2000 mJ·cm^−^^2^) (orange bars), (**B**) ascorbate peroxidase (APX) activity before stress (green bars) and 24 h after UV irradiation (2000 mJ·cm^−^^2^) (orange bars). Within a subgraph, values sharing common labels (letters) are not significantly different from each other (*p* > 0.001) (*n* = 9).

There is a strong relationship between SA and ROS content in plant tissues [5]. Moreover, SA can act as an inhibitor of some antioxidant enzymes, such as ascorbate peroxidase (APX) [53]. Therefore, we analyzed the level of H_2_O_2_ and APX activity in tested genotypes. Before stress, we found no statistical differences in the H_2_O_2_ content. Twenty-four hours after UV irradiation, the content of H_2_O_2_ increased in all tested genotypes in comparison with non-treated counterparts (Figure 4A). The UV-treated *lsd1* mutant showed significantly higher H_2_O_2_ level when compared with UV irradiated Col-0. Importantly, the mutation in *ICS1* or expression of *nahG* reversed this *lsd1*-specific accumulation of H_2_O_2_. The activity of APX before stress was higher in *NahG* plants and the *sid2*/*lsd1* mutant in comparison with the wild type (Figure 4B). Interestingly, after UV irradiation, the APX activity dropped significantly in *lsd1*, *NahG*, and *sid2*/*lsd1* in relation to non-treated counterparts. The *lsd1* mutant demonstrated the lowest APX activity, while *sid2*/*lsd1* and *NahG*/*lsd1* had comparable APX activity to Col-0 (Figure 4B). These results indicate that stress-induced redox changes in the *lsd1* mutant are modulated by SA content.

## 4. Discussion

PCD is an ultimate end of the cell cycle, which occurs in all living multicellular organisms. It is essential for the appropriate response of plants to biotic and abiotic stresses, but is also important in the regulation of growth and development [6,7,8]. One of the most important signaling molecules being engaged in this process is SA [49,54]. It was demonstrated that plants with higher SA accumulation exhibit greater potential for PCD [4,41]. Contrariwise, Arabidopsis genotypes with ameliorated SA metabolism or deregulation in SA synthesis exhibit better growth and fitness and higher seed yield [5,27]. However, SA does not act alone during PCD since other molecules such as ethylene and ROS are also involved [43,54,55,56].

Some of the best described Arabidopsis proteins in the context of PCD regulation are LSD1, EDS1, and PAD4 [5,15,22,42,57,58]. LSD1 is a negative regulator of PCD, suppressing EDS1 and PAD4 activities since the double mutants *eds1/lsd1* and *pad4/lsd1* demonstrate a reverted *lsd1*-specific phenotype in terms of SA, ethylene, and ROS accumulation and cell death [3,5,42,56].

It was previously found that deregulation of SA synthesis in the *lsd1* background reverts the cell death phenotype that occurs in response to biotic stress [57]. Notwithstanding, there is little information about the role of SA in response to short events of abiotic stresses such as high light, high temperature, or UV irradiation. Therefore, in the current work we focused on the role of SA accumulation in LSD1-regulated cell death triggered by UV. We obtained *sid2/lsd1* and *NahG*/*lsd1* plants in which the SA synthesis and metabolism were deregulated [24,27].

As expected [24,27,36,38], the dysfunctional mutation in *ICS1* or the expression of bacterial *nahG* in Arabidopsis strongly reduced SA accumulation in the double *sid2/lsd1* mutant and in the *NahG*/*lsd1* line both before stress and after UV irradiation. This result allowed us to continue our study of the role of SA in *lsd1*-specific cell death in response to UV stress.

UV-A, UV-B, and also UV-C irradiation were found to affect plants [58,59] and induce cell death [60,61]. The *lsd1* mutant is very susceptible to many abiotic stresses, such as high light [43], chilling [19], or UV-C [5,42].

In this study, we used UV A+B to induce cell death in Arabidopsis plants. It was demonstrated that UV A+B cause DNA damage, membrane disruption, protein crosslinking, and ROS formation [62]. Our results proved that before stress there was no difference in the phenotype of tested genotypes. These plants were grown in permissive short day and low light conditions that did not induce cell death in the *lsd1* mutant [15,43]. After UV irradiation, wild-type plants exhibited some visible changes, such as twisted leaves, and this effect was much more clear in the *lsd1* mutant, which is in line with previous studies [5,42]. What is particularly important is that *sid2/lsd1* and *NahG*/*lsd1* plants were in better shape than *lsd1* or even Col-0 after UV treatment.

The integrity of the cell membranes, tested using ion leakage, showed that the deregulation in SA synthesis/metabolism reverts the cell death phenotype of the *lsd1* mutant. Moreover, using TB staining we showed that after UV irradiation there were significantly more dead cells in the *lsd1* mutant than in Col-0 or *sid2/lsd1* and *NahG*/*lsd1*. It was shown previously that the mutation in *EDS1* or *PAD4* gene reverts the cell death phenotype in the *lsd1* mutant [5,17,42,55], but here we clearly show that SA acts as a crucial molecule in LSD1-dependent cell death signaling in response to abiotic stress.

The phenomenon of cell death phenotype reversal in the *lsd1* mutant via the deregulation in SA metabolism/synthesis can be caused by a significant reduction in *PR*-genes expression. PR proteins are necessary in hypersensitive response (HR) regulation, and both EDS1 and PAD4 are crucial in this pathway [63,64,65]. It was shown that the expression of bacterial nahG reduced *PR1* expression in response to biotic stress [66]. The *eds1/lsd1* or *pad4/lsd1* mutants lacking functional EDS1 or PAD4 proteins are not able to induce HR [20,67]. In the *lsd1* mutant, we found very high expression of *PR1*, *PR2*, and *PR5*, while in *sid2*/*lsd1* and *NahG*/*lsd1* plants, the *PR*-genes expression was similar to Col-0 or even lower. Higher expression of *PR2* in *sid2* may be related to the fact that *PR2* is not related to SA as much as *PR1* [68,69]. These results prove that SA is a crucial signaling molecule during abiotic stress response, that SA is necessary for the induction of *PR*-genes expression [70], and that the LSD1/EDS1/PAD4 hub [20,71] acts downstream of SA synthesis.

During PCD, not only does SA act as a signaling molecule, but ROS are also involved in this process [10,72,73,74,75]. Recently, a correlation between ROS, glutathione, ethylene, and SA content in plants has been found [3,5,55]. Moreover, it was proved that SA can affect the efficiency of the antioxidant system [1,53]. Therefore, the content of one of the ROS forms (H_2_O_2_) and the activity of enzymes involved in H_2_O_2_ scavenging (APX) were tested. In response to UV stress, all genotypes used in this study increased the content of H_2_O_2_ in their tissues. This effect was most significant in the *lsd1* mutant. It may be at least partially caused by higher SA level in the *lsd1* mutant since SA can inhibit the APX activity. APX is an important enzyme decomposing H_2_O_2_ into water [76,77] and is one of the most important enzymes in plant defense against oxidative stress [76]. The action of ROS during PCD is twofold—they induce cell damage but also act as signaling molecules [78]. Another ROS form, superoxide anion radical (O_2_^•−^), generated by respiratory burst oxidase homologs D and F (RBOHD/F), may antagonize pro-death signals induced during abiotic stress in Arabidopsis, since cell death was enhanced in *lsd1*/*rbohD* and *lsd1/rbohF* double mutants in comparison with the *lsd1* single mutant, implying that RBOHD/F function as suppressors of cell death in neighboring cells around sites of abiotic stress [74]. Taking into account that the lack of SA accumulation strongly correlates with the reversal of the *lsd1*-specific cell death phenotype in *sid2/lsd1* and *NahG*/*lsd1* plants, we postulate that SA induces H_2_O_2_ levels and acts as a negative regulator of the antioxidant system, which enhances PCD propagation.

In conclusion, our results show that SA is crucial in LSD1-dependent regulation of PCD and that deregulation of SA synthesis or metabolism inhibits the cell death phenotype in the *lsd1* background in response to abiotic stress.

## Figures and Tables

**Table 1:** Primers used for PCR and qPCR.

	Forward	Forward Tm [°C]	Reverse	Reverse Tm [°C]
*nahG (PCR)*	ACTCTGCCGCTACTCCCATA	63.7	CGAGCCCTAGGTACATCTGC	63.7
*sid2 (PCR)*	TGTCTGCAGTGAAGCTTTGG	64.2	CGAAGAAATGAAGAGCTTGGA	63.3
*nahG* (qPCR)	CACCGGGCGGATTTCAT	67	CCCGAATTGGGCGATACC	61.1
*5-FCL* (ref for qPCR)	GCAAACTCAATGAACATTTTGG	63.1	GATCGGTTCATCTGCTTGC	63.5
*PP2AA2* (ref for qPCR)	TAACGTGGCCAAAATGATGC	65.6	GTTCTCCACAACCGCTTGGT	66.3
*PR1* (qPCR)	TTCTTCCCTCGAAAGCTCAA	63.9	GCCTGGTTGTGAACCCTTAG	63.4
*PR2* (qPCR)	TCTCCCTTGCTCGTGAATCT	63.9	CGTGTCTCCCATGTAGCTGA	64
*PR5* (qPCR)	CGTACAGGCTGCAACTTTGA	64	CTTAGACCGCCACAGTCTCC	63.8

## Data Availability

The datasets generated during and/or analyzed during the current study are available from the corresponding author on reasonable request.

## References

[1] Karpiński S., Szechyńska-Hebda M., Wituszyńska W., Burdiak P. (2013). Light Acclimation, Retrograde Signalling, Cell Death and Immune Defences in Plants. Plant Cell Environ..

[2] Mühlenbock P., Szechynska-Hebda M., Plaszczyca M., Baudo M., Mateo A., Mullineaux P.M., Parker J.E., Karpinska B., Karpinski S. (2008). Chloroplast Signaling and LESION SIMULATING DISEASE1 Regulate Crosstalk between Light Acclimation and Immunity in Arabidopsis. Plant Cell.

[3] Mühlenbock P., Plaszczyca M., Plaszczyca M., Mellerowicz E., Karpinski S. (2007). Lysigenous Aerenchyma Formation in Arabidopsis Is Controlled by LESION SIMULATING DISEASE1. Plant Cell.

[4] Wituszyńska W., Ślesak I., Vanderauwera S., Szechyńska-Hebda M., Kornaś A., Kelen K.V.D., Mühlenbock P., Karpińska B., Maćkowski S., Breusegem F.V. (2013). LESION SIMULATING DISEASE1, ENHANCED DISEASE SUSCEPTIBILITY1, and PHYTOALEXIN DEFICIENT4 Conditionally Regulate Cellular Signaling Homeostasis, Photosynthesis, Water Use Efficiency, and Seed Yield in Arabidopsis. Plant Physiol..

[5] Bernacki M.J., Czarnocka W., Rusaczonek A., Witoń D., Kęska S., Czyż J., Szechyńska-Hebda M., Karpiński S. (2018). LSD1, EDS1 and PAD4-Dependent Conditional Correlation among Salicylic Acid, Hydrogen Peroxide, Water Use Efficiency, and Seed Yield in Arabidopsis Thaliana. Physiol. Plant..

[6] Wituszynska W., Karpinski S., Vahdati K. (2013). Programmed Cell Death as a Response to High Light, UV and Drought Stress in Plants. Abiotic Stress—Plant Responses and Applications in Agriculture.

[7] Fuchs Y., Steller H. (2015). Live to Die Another Way: Modes of Programmed Cell Death and the Signals Emanating from Dying Cells. Nat. Rev. Mol. Cell Biol..

[8] Elmore S. (2007). Apoptosis: A Review of Programmed Cell Death. Toxicol. Pathol..

[9] Sychta K., Słomka A., Kuta E. (2021). Insights into Plant Programmed Cell Death Induced by Heavy Metals-Discovering a Terra Incognita. Cells.

[10] Czarnocka W., Karpiński S. (2018). Friend or Foe? Reactive Oxygen Species Production, Scavenging and Signaling in Plant Response to Environmental Stresses. Free Radic. Biol. Med..

[11] van Doorn W.G., Woltering E.J. (2005). Many Ways to Exit? Cell Death Categories in Plants. Trends Plant Sci..

[12] Spoel S.H., Dong X. (2012). How Do Plants Achieve Immunity? Defence without Specialized Immune Cells. Nat. Rev. Immunol..

[13] Szechyńska-Hebda M., Kruk J., Górecka M., Karpińska B., Karpiński S. (2010). Evidence for Light Wavelength-Specific Photoelectrophysiological Signaling and Memory of Excess Light Episodes in Arabidopsis. Plant Cell.

[14] Górecka M., Lewandowska M., Dąbrowska-Bronk J., Białasek M., Barczak-Brzyżek A., Kulasek M., Mielecki J., Kozłowska-Makulska A., Gawroński P., Karpiński S. (2020). Photosystem II 22kDa Protein Level—A Prerequisite for Excess Light-Inducible Memory, Cross-Tolerance to UV-C and Regulation of Electrical Signalling. Plant Cell Environ..

[15] Dietrich R.A., Richberg M.H., Schmidt R., Dean C., Dangl J.L. (1997). A Novel Zinc Finger Protein Is Encoded by the Arabidopsis LSD1 Gene and Functions as a Negative Regulator of Plant Cell Death. Cell.

[16] Czarnocka W., Van Der Kelen K., Willems P., Szechyńska-Hebda M., Shahnejat-Bushehri S., Balazadeh S., Rusaczonek A., Mueller-Roeber B., Van Breusegem F., Karpiński S. (2017). The Dual Role of LESION SIMULATING DISEASE 1 as a Condition-Dependent Scaffold Protein and Transcription Regulator. Plant Cell Environ..

[17] Rustérucci C., Aviv D.H., Holt B.F., Dangl J.L., Parker J.E. (2001). The Disease Resistance Signaling Components EDS1 and PAD4 Are Essential Regulators of the Cell Death Pathway Controlled by LSD1 in Arabidopsis. Plant Cell.

[18] Chai T., Zhou J., Liu J., Xing D. (2015). LSD1 and HY5 Antagonistically Regulate Red Light Induced-Programmed Cell Death in Arabidopsis. Front Plant Sci..

[19] Huang X., Li Y., Zhang X., Zuo J., Yang S. (2010). The Arabidopsis LSD1 Gene Plays an Important Role in the Regulation of Low Temperature-Dependent Cell Death. New Phytol..

[20] Feys B.J., Moisan L.J., Newman M.-A., Parker J.E. (2001). Direct Interaction between the Arabidopsis Disease Resistance Signaling Proteins, EDS1 and PAD4. EMBO J..

[21] Shah J. (2003). The Salicylic Acid Loop in Plant Defense. Curr. Opin. Plant Biol..

[22] Wiermer M., Feys B.J., Parker J.E. (2005). Plant Immunity: The EDS1 Regulatory Node. Curr. Opin. Plant Biol..

[23] Vlot A.C., Dempsey D.A., Klessig D.F. (2009). Salicylic Acid, a Multifaceted Hormone to Combat Disease. Annu. Rev. Phytopathol..

[24] Wildermuth M.C., Dewdney J., Wu G., Ausubel F.M. (2001). Isochorismate Synthase Is Required to Synthesize Salicylic Acid for Plant Defence. Nature.

[25] Duan C., Yu J., Bai J., Zhu Z., Wang X. (2014). Induced Defense Responses in Rice Plants against Small Brown Planthopper Infestation. Crop J..

[26] Lefevere H., Bauters L., Gheysen G. (2020). Salicylic Acid Biosynthesis in Plants. Front Plant Sci.

[27] Abreu M.E., Munné-Bosch S. (2009). Salicylic Acid Deficiency in NahG Transgenic Lines and Sid2 Mutants Increases Seed Yield in the Annual Plant Arabidopsis Thaliana. J. Exp. Bot..

[28] Hao Q., Wang W., Han X., Wu J., Lyu B., Chen F., Caplan A., Li C., Wu J., Wang W. (2018). Isochorismate-Based Salicylic Acid Biosynthesis Confers Basal Resistance to Fusarium Graminearum in Barley. Mol. Plant Pathol..

[29] Catinot J., Buchala A., Abou-Mansour E., Métraux J.-P. (2008). Salicylic Acid Production in Response to Biotic and Abiotic Stress Depends on Isochorismate in Nicotiana Benthamiana. FEBS Lett..

[30] Mercado-Blanco J., van der Drift K.M.G.M., Olsson P.E., Thomas-Oates J.E., van Loon L.C., Bakker P.A.H.M. (2001). Analysis of the PmsCEAB Gene Cluster Involved in Biosynthesis of Salicylic Acid and the Siderophore Pseudomonine in the Biocontrol Strain Pseudomonas Fluorescens WCS374. J. Bacteriol..

[31] Torrens-Spence M.P., Bobokalonova A., Carballo V., Glinkerman C.M., Pluskal T., Shen A., Weng J.-K. (2019). PBS3 and EPS1 Complete Salicylic Acid Biosynthesis from Isochorismate in Arabidopsis. Mol. Plant.

[32] Rekhter D., Lüdke D., Ding Y., Feussner K., Zienkiewicz K., Lipka V., Wiermer M., Zhang Y., Feussner I. (2019). Isochorismate-Derived Biosynthesis of the Plant Stress Hormone Salicylic Acid. Science.

[33] Nawrath C., Métraux J.P. (1999). Salicylic Acid Induction-Deficient Mutants of Arabidopsis Express PR-2 and PR-5 and Accumulate High Levels of Camalexin after Pathogen Inoculation. Plant Cell.

[34] Yokoo S., Inoue S., Suzuki N., Amakawa N., Matsui H., Nakagami H., Takahashi A., Arai R., Katou S. (2018). Comparative Analysis of Plant Isochorismate Synthases Reveals Structural Mechanisms Underlying Their Distinct Biochemical Properties. Biosci. Rep..

[35] Garcion C., Lohmann A., Lamodière E., Catinot J., Buchala A., Doermann P., Métraux J.-P. (2008). Characterization and Biological Function of the ISOCHORISMATE SYNTHASE2 Gene of Arabidopsis. Plant Physiol..

[36] Morse A.M., Tschaplinski T.J., Dervinis C., Pijut P.M., Schmelz E.A., Day W., Davis J.M. (2007). Salicylate and Catechol Levels Are Maintained in NahG Transgenic Poplar. Phytochemistry.

[37] Miura K., Okamoto H., Okuma E., Shiba H., Kamada H., Hasegawa P.M., Murata Y. (2013). SIZ1 Deficiency Causes Reduced Stomatal Aperture and Enhanced Drought Tolerance via Controlling Salicylic Acid-Induced Accumulation of Reactive Oxygen Species in Arabidopsis. Plant J..

[38] Gawroński P., Witoń D., Vashutina K., Bederska M., Betliński B., Rusaczonek A., Karpiński S. (2014). Mitogen-Activated Protein Kinase 4 Is a Salicylic Acid-Independent Regulator of Growth but Not of Photosynthesis in Arabidopsis. Mol. Plant.

[39] Devadas S.K., Raina R. (2002). Preexisting Systemic Acquired Resistance Suppresses Hypersensitive Response-Associated Cell Death in Arabidopsishrl1 Mutant. Plant Physiol..

[40] Nawrath C., Heck S., Parinthawong N., Métraux J.-P. (2002). EDS5, an Essential Component of Salicylic Acid–Dependent Signaling for Disease Resistance in Arabidopsis, Is a Member of the MATE Transporter Family. Plant Cell.

[41] Radojičić A., Li X., Zhang Y. (2018). Salicylic Acid: A Double-Edged Sword for Programed Cell Death in Plants. Front. Plant Sci..

[42] Wituszyńska W., Szechyńska-Hebda M., Sobczak M., Rusaczonek A., Kozłowska-Makulska A., Witoń D., Karpiński S. (2015). Lesion Simulating Disease 1 and Enhanced Disease Susceptibility 1 Differentially Regulate UV-C-Induced Photooxidative Stress Signalling and Programmed Cell Death in Arabidopsis Thaliana. Plant Cell Environ..

[43] Mateo A., Mühlenbock P., Rustérucci C., Chang C.C.-C., Miszalski Z., Karpinska B., Parker J.E., Mullineaux P.M., Karpinski S. (2004). LESION SIMULATING DISEASE 1 Is Required for Acclimation to Conditions That Promote Excess Excitation Energy. Plant Physiol..

[44] Friedrich L., Vernooij B., Gaffney T., Morse A., Ryals J. (1995). Characterization of Tobacco Plants Expressing a Bacterial Salicylate Hydroxylase Gene. Plant Mol. Biol..

[45] Witoń D., Gawroński P., Czarnocka W., Ślesak I., Rusaczonek A., Sujkowska-Rybkowska M., Bernacki M.J., Dąbrowska-Bronk J., Tomsia N., Szechyńska-Hebda M. (2016). Mitogen Activated Protein Kinase 4 (MPK4) Influences Growth in *Populus tremula* L. × *tremuloides*. Environ. Exp. Bot..

[46] Rusaczonek A., Czarnocka W., Kacprzak S., Witoń D., Ślesak I., Szechyńska-Hebda M., Gawroński P., Karpiński S. (2015). Role of Phytochromes A and B in the Regulation of Cell Death and Acclimatory Responses to UV Stress in Arabidopsis Thaliana. J. Exp. Bot..

[47] Bernacki M.J., Czarnocka W., Witoń D., Rusaczonek A., Szechyńska-Hebda M., Ślesak I., Dąbrowska-Bronk J., Karpiński S. (2018). ENHANCED DISEASE SUSCEPTIBILITY 1 (EDS1) Affects Development, Photosynthesis, and Hormonal Homeostasis in Hybrid Aspen (*Populus tremula* L. × *P. tremuloides*). J. Plant Physiol..

[48] Bhar A., Chatterjee M., Gupta S., Das S. (2018). Salicylic Acid Regulates Systemic Defense Signaling in Chickpea During Fusarium Oxysporum f. Sp. Ciceri Race 1 Infection. Plant Mol. Biol. Rep..

[49] Delaney T.P., Uknes S., Vernooij B., Friedrich L., Weymann K., Negrotto D., Gaffney T., Gut-Rella M., Kessmann H., Ward E. (1994). A Central Role of Salicylic Acid in Plant Disease Resistance. Science.

[50] Alvarez M.E., Pennell R.I., Meijer P.J., Ishikawa A., Dixon R.A., Lamb C. (1998). Reactive Oxygen Intermediates Mediate a Systemic Signal Network in the Establishment of Plant Immunity. Cell.

[51] Czarnocka W., Fichman Y., Bernacki M., Różańska E., Sańko-Sawczenko I., Mittler R., Karpiński S. (2020). FMO1 Is Involved in Excess Light Stress-Induced Signal Transduction and Cell Death Signaling. Cells.

[52] Morris K., Mackerness S.A.H., Page T., John C.F., Murphy A.M., Carr J.P., Buchanan-Wollaston V. (2000). Salicylic Acid Has a Role in Regulating Gene Expression during Leaf Senescence. Plant J..

[53] Durner J., Klessig D.F. (1995). Inhibition of Ascorbate Peroxidase by Salicylic Acid and 2,6-Dichloroisonicotinic Acid, Two Inducers of Plant Defense Responses. Proc. Natl. Acad. Sci. USA.

[54] Alvarez M.E., Lam E., Fukuda H., Greenberg J. (2000). Salicylic acid in the machinery of hypersensitive cell death and disease resistance. Programmed Cell Death in Higher Plants.

[55] Mateo A., Funck D., Mühlenbock P., Kular B., Mullineaux P.M., Karpinski S. (2006). Controlled Levels of Salicylic Acid Are Required for Optimal Photosynthesis and Redox Homeostasis. J. Exp. Bot..

[56] Karpinski S., Wingsle G., Karpinska B., Hällgren J.-E., Aro E.-M., Andersson B. (2001). Redox Sensing of Photooxidative Stress and Acclimatory Mechanisms in Plants. Regulation of Photosynthesis.

[57] Aviv D.H., Rustérucci C., Iii B.F.H., Dietrich R.A., Parker J.E., Dangl J.L. (2002). Runaway Cell Death, but Not Basal Disease Resistance, in Lsd1 Is SA- and NIM1/NPR1-Dependent. Plant J..

[58] Kalbina I., Li S., Kalbin G., Björn L.O., Strid Å. (2008). Two Separate UV-B Radiation Wavelength Regions Control Expression of Different Molecular Markers in Arabidopsis Thaliana. Funct. Plant Biol..

[59] Britt A.B. (2004). Repair of DNA Damage Induced by Solar UV. Photosynth. Res..

[60] Rusaczonek A., Czarnocka W., Willems P., Sujkowska-Rybkowska M., Van Breusegem F., Karpiński S. (2021). Phototropin 1 and 2 Influence Photosynthesis, UV-C Induced Photooxidative Stress Responses, and Cell Death. Cells.

[61] Nawkar G.M., Maibam P., Park J.H., Sahi V.P., Lee S.Y., Kang C.H. (2013). UV-Induced Cell Death in Plants. Int. J. Mol. Sci..

[62] Dotto M., Casati P. (2017). Developmental Reprogramming by UV-B Radiation in Plants. Plant Sci..

[63] Morel J.-B., Dangl J.L. (1997). The Hypersensitive Response and the Induction of Cell Death in Plants. Cell Death Differ..

[64] Cui H., Gobbato E., Kracher B., Qiu J., Bautor J., Parker J.E. (2017). A Core Function of EDS1 with PAD4 Is to Protect the Salicylic Acid Defense Sector in Arabidopsis Immunity. New Phytol..

[65] Falk A., Feys B.J., Frost L.N., Jones J.D.G., Daniels M.J., Parker J.E. (1999). EDS1, an Essential Component of R Gene-Mediated Disease Resistance in Arabidopsis Has Homology to Eukaryotic Lipases. Proc. Natl. Acad. Sci. USA.

[66] Zou B., Jia Z., Tian S., Wang X., Gou Z., Lü B., Dong H. (2013). AtMYB44 Positively Modulates Disease Resistance to Pseudomonas Syringae through the Salicylic Acid Signalling Pathway in Arabidopsis. Funct. Plant Biol..

[67] Bernacki M.J., Czarnocka W., Zaborowska M., Różańska E., Labudda M., Rusaczonek A., Witoń D., Karpiński S. (2020). EDS1-Dependent Cell Death and the Antioxidant System in Arabidopsis Leaves Is Deregulated by the Mammalian Bax. Cells.

[68] Reuber T.L., Plotnikova J.M., Dewdney J., Rogers E.E., Wood W., Ausubel F.M. (1998). Correlation of Defense Gene Induction Defects with Powdery Mildew Susceptibility in Arabidopsis Enhanced Disease Susceptibility Mutants. Plant J..

[69] Thibaud M.-C., Gineste S., Nussaume L., Robaglia C. (2004). Sucrose Increases Pathogenesis-Related PR-2 Gene Expression in Arabidopsis Thaliana through an SA-Dependent but NPR1-Independent Signaling Pathway. Plant Physiol. Biochem..

[70] Mullineaux P.M., Rausch T. (2005). Glutathione, Photosynthesis and the Redox Regulation of Stress-Responsive Gene Expression. Photosynth Res..

[71] Feys B.J., Wiermer M., Bhat R.A., Moisan L.J., Medina-Escobar N., Neu C., Cabral A., Parker J.E. (2005). Arabidopsis SENESCENCE-ASSOCIATED GENE101 Stabilizes and Signals within an ENHANCED DISEASE SUSCEPTIBILITY1 Complex in Plant Innate Immunity. Plant Cell.

[72] Møller I.M., Sweetlove L.J. (2010). ROS Signalling—Specificity Is Required. Trends Plant Sci..

[73] Bailey-Serres J., Mittler R. (2006). The Roles of Reactive Oxygen Species in Plant Cells. Plant Physiol..

[74] Torres M.A., Jones J.D.G., Dangl J.L. (2005). Pathogen-Induced, NADPH Oxidase–Derived Reactive Oxygen Intermediates Suppress Spread of Cell Death in Arabidopsis Thaliana. Nat. Genet..

[75] Kim C., Apel K. (2013). Singlet Oxygen-Mediated Signaling in Plants: Moving from Flu to Wild Type Reveals an Increasing Complexity. Photosynth Res..

[76] Foyer C.H., Noctor G. (2009). Redox Regulation in Photosynthetic Organisms: Signaling, Acclimation, and Practical Implications. Antioxid. Redox. Signal..

[77] Mittler R. (2002). Oxidative Stress, Antioxidants and Stress Tolerance. Trends Plant Sci..

[78] Scherz-Shouval R., Elazar Z. (2011). Regulation of Autophagy by ROS: Physiology and Pathology. Trends Biochem. Sci..

